# Supplementary material for: Splice-Junction-Based Mapping of Alternative Isoforms in the Human Proteome
Source: Cell Rep. Author manuscript; Available in PMC 2020 Jan 15. (PMC6961840; doi:10.1016/j.celrep.2019.11.026)
Supplement: 2 [file NIHMS1546469-supplement-2.zip › DF1/PXD010154/Colon/Colon_15_STXBP2_LEDIALR.pdf]

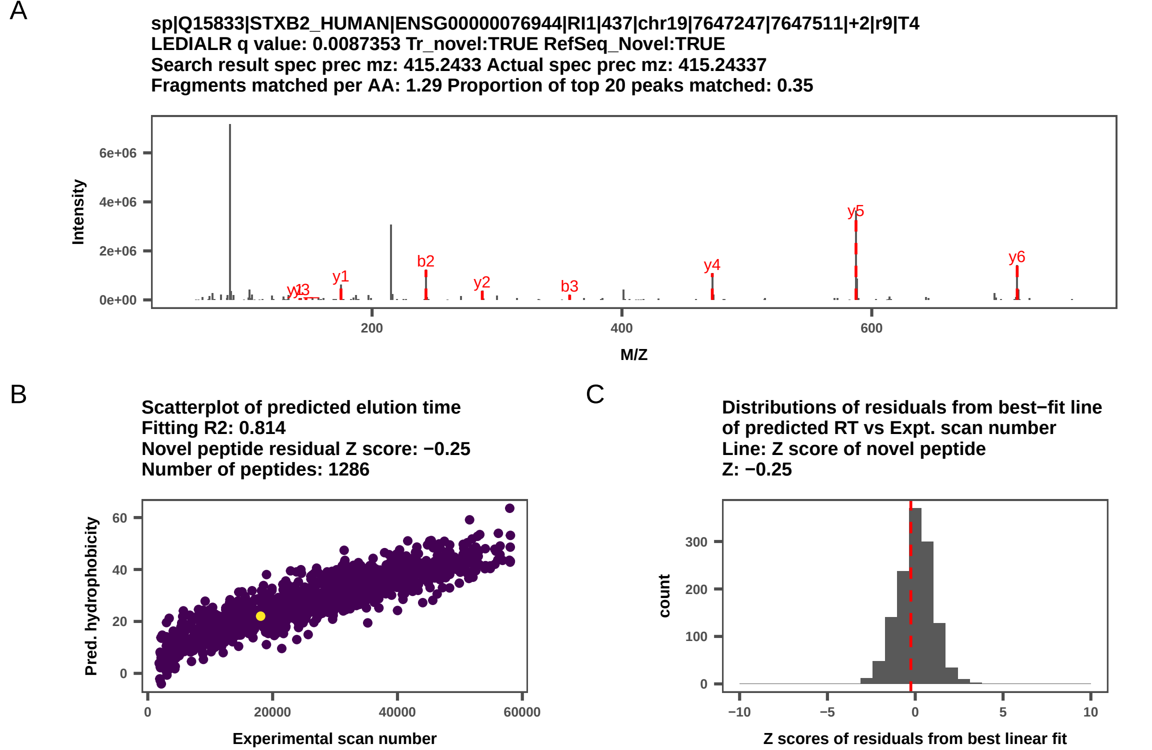
A
sp|Q15833|STXB2_HUMAN|ENSG00000076944|RI1|437|chr19|7647247|7647511|+2|r9|T4
LEDIALR q value: 0.0087353 Tr_novel:TRUE RefSeq_Novel:TRUE
Search result spec prec mz: 415.2433 Actual spec prec mz: 415.24337
Fragments matched per AA: 1.29 Proportion of top 20 peaks matched: 0.35
Intensity
6e+06
4e+06
2e+06
0e+00
y1
b2
y2
b3
y4
y5
y6
200
400
600
M/Z
B
Scatterplot of predicted elution time
Fitting R2: 0.814
Novel peptide residual Z score: −0.25
Number of peptides: 1286
Pred. hydrophobicity
60
40
20
0
0
20000
40000
60000
Experimental scan number
C
Distributions of residuals from best−fit line
of predicted RT vs Expt. scan number
Line: Z score of novel peptide
Z: −0.25
count
300
200
100
0
−10
−5
0
5
10
Z scores of residuals from best linear fit
